# Prognostic Role of the Modified Frailty Index in Octogenarians Undergoing Minimally Invasive Aortic Valve Replacement

**DOI:** 10.3390/jcm14144833

**Published:** 2025-07-08

**Authors:** Beatrice Bacchi, Francesco Cabrucci, Dario Petrone, Giulia Bessi, Tommaso Pacini, Aleksander Dokollari, Massimo Bonacchi

**Affiliations:** 1 Cardiac Surgery Unit, Department of Experimental and Clinical Medicine, University of Florence, 50121 Firenze, Italy; beatrice.bacchi@unifi.it (B.B.); dario.petrone@unifi.it (D.P.); giulia.bessi@unifi.it (G.B.); tommaso.pacini1@edu.unifi.it (T.P.); 2 Department of Cardiac Surgery Research, Lankenau Institute for Medical Research, Wynnewood, PA 19096, USA; cabruccif@mlhs.org; 3 Cardiac Surgery Department, St. Boniface Hospital, University of Manitoba, Winnipeg, MB R2H 2A6, Canada; aleksanderdokollari2@gmail.com

**Keywords:** frailty, modified Frailty Index, aortic valve replacement, minimally invasive surgery, octogenarians, risk stratification

## Abstract

**Objectives:** Frailty is increasingly recognized as a key determinant of surgical risk in elderly patients undergoing aortic valve replacement (AVR). This study aimed to evaluate the prognostic value of the modified Frailty Index (mFI) in a homogeneous cohort of octogenarians undergoing minimally invasive surgical AVR, to enhance risk stratification and guide surgical decision-making. **Methods:** We retrospectively analyzed 67 patients aged ≥ 80 years (mean 84.1 ± 3.2) who underwent isolated minimally invasive AVR. The mFI was calculated preoperatively using standardized clinical variables. Primary outcomes included 30-day mortality and perioperative complications; long-term survival was also assessed. Receiver operating characteristic (ROC) curves identified optimal mFI cut-offs. Kaplan-Meier and Cox regression analyses were used to evaluate survival and predictors of mortality. **Results:** The mFI demonstrated a strong prognostic accuracy. An mFI > 0.455 predicted 30-day mortality with 81.8% sensitivity and 88.4% specificity (AUC = 0.888, *p* < 0.001), while an mFI > 0.273 predicted perioperative complications (AUC = 0.818, *p* < 0.001). During a median follow-up of 51.8 ± 36.4 months, 24 patients (45.3%) died. One-year survival was 83.7%. The mFI > 0.455 was the strongest independent predictor of early mortality (HR 6.34, *p* = 0.001); mFI > 0.273, HFpEF with NT-proBNP > 1000 pg/mL, and chronic kidney disease were predictors of long-term mortality. **Conclusions:** The mFI is a simple, reproducible tool that reliably predicts early and late outcomes in very elderly patients undergoing minimally invasive AVR. Integrating frailty into preoperative evaluation may improve patient selection by prioritizing physiological over chronological age.

## 1. Introduction

Aortic stenosis (AS) in very elderly patients (≥80 years old) represents a growing clinical challenge in an era increasingly dominated by transcatheter approaches [1,2]. While transcatheter aortic valve implantation (TAVI) has emerged as the first choice in older and high-risk patients [3,4], surgical aortic valve replacement (SAVR) remains a valuable option in selected cases, particularly where anatomical or technical factors preclude a transcatheter approach [5]. In such patients, careful preoperative assessment becomes essential to balance the potential benefits of surgery against the risk of futility [6].

The concept of futility has gained increasing relevance in decision-making for elderly patients undergoing AVR [7,8]. Evidence from major TAVI trials and registries suggests that up to 30–50% of patients may experience futility at one year, particularly those with irreversible symptoms, severe comorbidities, or impaired functional status [9,10]. Notably, advanced age alone does not equate to futility, as even patients over 85 years of age may derive significant benefit when appropriately selected [11].

Among the non-cardiac predictors of poor outcome, frailty has emerged as a central determinant of both procedural risk and long-term recovery [12,13]. Despite the growing recognition of its prognostic value, no universally accepted frailty score has been integrated into routine clinical practice [14,15]. Various tools—ranging from physical performance tests to biochemical and radiographic markers—have been proposed, each with specific limitations in terms of feasibility, reproducibility, or availability [14].

The modified Frailty Index (mFI) is a validated, comorbidity-based score [16]. Its simplicity, reliance on routinely available data, and ease of retrospective and prospective application make it particularly suitable for surgical settings [17]. In this study, we aimed to evaluate the prognostic role of the mFI in a homogeneous cohort of octogenarians undergoing minimally invasive SAVR, to enhance risk stratification and identify patients most likely to benefit from surgical intervention.

## 2. Materials and Methods

### 2.1. Ethics

All data were anonymized prior to analysis. The study was conducted in accordance with the Declaration of Helsinki. Ethical review and approval were waived for this study due to the nature of the study.

### 2.2. Patients

This retrospective multi-institutional observational cohort study analyzed prospectively collected data from 67 patients (mean age 84.1 ± 3.2 years) who underwent minimally invasive isolated AVR for severe aortic valve dysfunction from 2009 to 2022. The patients were not considered for a TAVI due to anatomical or technical contraindications, such as small aortic annulus, severely calcified or tortuous iliofemoral vessels, extensive aortic root calcification, bicuspid aortic valves with asymmetric calcification, or predominant aortic regurgitation. Additionally, part of the study cohort was treated during a time period in which the TAVI was not yet routinely available.

All procedures were conducted under general anesthesia following the same anesthetic regimen and monitoring. Anesthesia was induced with intravenous propofol (Fresenius Kabi, Lake Zurich, IL, USA), fentanyl (Hameln Pharma, Hameln, Germany), and rocuronium bromide (Merck & Co., Inc., Kenilworth, NJ, USA). Maintainance of anesthesia was achieved using a balanced technique with sevoflurane (AbbVie Inc., North Chicago, IL, USA) in an oxygen–air mixture. The depth of anesthesia was continuously monitored and adjusted intraoperatively, and invasive arterial and central venous pressures were continuously recorded. Intraoperative monitoring included 5-lead ECG, pulse oximetry, capnography, urinary output, nasopharyngeal temperature, and continuous cardiac output monitoring.

AVR was performed through either an upper ministernotomy (MS), performed through a reversed-C or reversed-L incision or a right anterior mini-thoracotomy (MT), in the second or third intercostal space, depending on anatomical characteristics [18]. After pericardial opening and exposure of the aortic root, a standard cannulation of the ascending aorta and right atrium was performed in the MS, when feasible. In particular, peripheral cannulation was used in 58 patients (86.6%), while central (direct aortic and right atrial) cannulation was adopted in the remaining 9 patients (13.4%). In patients with a documented history of PAD—defined as intermittent claudication, prior peripheral vascular intervention, or angiographically confirmed arterial stenosis—the choice of cannulation strategy was based on preoperative CT angiographic assessment. Among the 16 patients with PAD, 9 underwent the central cannulation due to an unfavorable peripheral anatomy, while 7 safely underwent the peripheral cannulation following a confirmation of adequate femoral vessel characteristics.

Aortic cross-clamping was performed directly through the thoracotomy incision or through the mini-sternotomy access. Myocardial protection was achieved using repeated doses of warm anterograde blood cardioplegia administered every 20–25 min, either into the aortic root or selectively into the coronary ostia.

The valve implantation technique was identical in both surgical approaches. Intraoperative transoesophageal echocardiography (TEE) was systematically employed to evaluate the cardiac function, confirm the prosthesis positioning and competence, ensure effective air evacuation, and exclude perivalvular leaks. While prosthetic valve selection was based on patient anatomy and surgeon preference, the most commonly implanted valves included stented bioprostheses (Perimount Magna Ease, Edwards Lifesciences, Irvine, CA, USA) and sutureless valves (Perceval S, Corcym, Saluggia, Italy). Temporary epicardial pacing wires were placed, and chest drains were positioned in the pericardial space and/or pleural cavity, depending on the approach used. Neuromuscular blockade was reversed at the end of surgery with sugammadex (MSD, Kenilworth, NJ, USA).

After surgery, all patients were transferred to the intensive care unit (ICU), where ventilatory support was provided via intermittent mandatory ventilation with a tidal volume of 8–10 mL/kg, a respiratory rate of 12–14 breaths per minute, a pressure support of 10–20 cmH_2_O, and a positive end-expiratory pressure (PEEP) of 3–5 cmH_2_O. Arterial blood gases were measured at 1 and 4 h before and after extubation. Postoperative chest X-rays were obtained daily. Pain was assessed using a standardized 5-point scale and managed with intravenous morphine and nonsteroidal anti-inflammatory drugs (NSAIDs) when scores were ≥3. Patients were extubated once hemodynamic and respiratory parameters were stable, and standard weaning protocols were followed [19].

Despite standardization of the anesthetic drugs, this was a multicenter study, and therefore, heterogeneity in monitoring and surgical equipment was present across institutions. While core intraoperative principles were consistent, individual centers used different devices depending on institutional availability. Examples include TEE systems such as the Vivid E95 (GE Healthcare, Chicago, IL, USA) and EPIQ CVx (Philips Medical Systems, Andover, MA, USA); arterial pressure lines from Edwards Lifesciences (Irvine, CA, USA) or Becton Dickinson (Franklin Lakes, NJ, USA); and central venous catheters such as Arrow triple-lumen catheters (Teleflex, Wayne, PA, USA).

Frailty was assessed using the Modified Frailty Index (mFI), based on 11 variables derived from the Canadian Study of Health and Aging Frailty Index. The variables include a history of diabetes mellitus, hypertension requiring medication, congestive heart failure, chronic obstructive pulmonary disease, myocardial infarction, percutaneous coronary intervention or cardiac surgery, cerebrovascular accident or transient ischemic attack, impaired sensorium, functional dependence, and history of peripheral vascular disease. Each variable present was assigned a value of 1, and the final mFI score was calculated by dividing the number of positive variables by the total number of variables assessed, resulting in a score ranging from 0 to 1.

The baseline demographic and preoperative characteristics are summarized in Table 1, while the perioperative and postoperative variables are reported in Table 2. The mean operative time was 212 ± 61.4 min, with cardiopulmonary bypass and aortic cross-clamp times of 61.1 ± 41.4 min and 53.7 ± 32.6 min, respectively. A majority of patients received stented tissue valves, followed by stentless and sutureless valves. Conversion to full sternotomy occurred in two cases. Postoperative complications included new-onset atrial fibrillation (22%), pacemaker implantation (4.5%), perioperative stroke (3%), and myocardial infarction (1.5%). Blood transfusions were required in 31% of patients, and re-exploration for bleeding was necessary in two cases. Groin access complications were rare, including isolated infections, lymphoceles, and femoral pseudoaneurysms. Respiratory complications included significant pulmonary atelectasis (15%) and prolonged ventilation >12 h in 7.5% of patients. The average ICU stay was 18.3 h, with prolonged ICU stay (>3 days) occurring in 6% of patients. Pain scores decreased progressively from ICU discharge (mean 4.39) to hospital discharge (1.85) and at one-month follow-up (1.37). The 30-day mortality rate was 9%, with four cases attributed to cardiac causes. Several variables, including high frailty index, elevated NT-proBNP, and prolonged ventilation or ICU stay, were significantly associated with adverse outcomes.

The postoperative follow-up was performed in-hospital after one month and subsequently in the event of complications. In the absence of complications, a long-term follow-up was conducted via telephone or email at 1, 3, 5, 10, and 20 years postoperatively. All data were entered into a dedicated institutional database.

The mean follow-up was 51.8 ± 36.4 months (IQR 0–82 months).

### 2.3. Definitions and Endpoints

In calculating the mFI, comorbidities were defined according to standardized clinical criteria to ensure consistency and reproducibility. Specifically, peripheral artery disease (PAD) was defined as a history of intermittent claudication, prior peripheral vascular intervention (angioplasty or surgery), or angiographically confirmed stenosis. Chronic kidney disease (CKD) was defined as an estimated glomerular filtration rate (eGFR) < 60 mL/min/1.73 m^2^ or the requirement for chronic dialysis, in accordance with KDIGO guidelines. Chronic obstructive pulmonary disease (COPD) was defined as a documented diagnosis of chronic bronchitis or emphysema by a pulmonologist, and/or consistent pulmonary function tests, with or without the need for long-term oxygen therapy.

The primary endpoints were as follows: (1) identification of the optimal mFI cutoff value for predicting the 30-day mortality; and (2) identification of the optimal mFI cutoff value for predicting the perioperative complications. The perioperative complications were defined as adverse events occurring within 30 days of surgery or during the index hospitalization. These included (i) stroke, defined as any acute neurological deficit lasting >24 h that is of confirmed cerebrovascular origin, diagnosed by a neurologist, and supported by neuroimaging (CT or MRI); (ii) acute kidney failure (AKF), defined as a postoperative serum creatinine level > 200 µmol/L or the need for dialysis; (iii) prolonged ventilatory support, defined as pulmonary insufficiency requiring mechanical ventilation for more than 24 h; (iv) significant pleural effusion, defined as fluid accumulation necessitating surgical drainage; and (v) clinically significant pneumothorax or subcutaneous emphysema requiring surgical intervention. Secondary endpoints included (1) identification of predictors of short-term outcomes and (2) identification of predictors of long-term outcomes.

### 2.4. Statistical Analysis

This was an observational study based on a pre-specified analysis of prospectively collected clinical data. Descriptive statistics were used to summarize baseline characteristics. Categorical variables are presented as counts and percentages, whereas continuous variables are expressed as mean ± standard deviation (SD) or median with interquartile range (IQR), contingent upon the result of the Shapiro-Wilk test for normality.

The mFI was analyzed both as a continuous and a categorical variable. Receiver operating characteristic (ROC) curves were constructed to evaluate the discriminative ability of the mFI for a 30-day mortality, long-term mortality, and postoperative complications. The optimal cut-off values were identified based on the maximum Youden Index (sensitivity + specificity − 1).

A survival analysis was performed using the Kaplan-Meier method. Multivariable Cox proportional hazards regression models were applied to identify independent predictors of both short- and long-term mortality, including clinically relevant covariates and those with statistical significance in the univariable analysis.

All statistical analyses were performed using IBM^®^ SPSS^®^ Statistics (version 25.0; IBM Corp., Armonk, NY, USA) and R software (version 3.6.3; R Foundation for Statistical Computing, Vienna, Austria), employing the ‘Amelia’ package for missing data imputation and the ‘E-Value’ package for sensitivity analyses. A two-sided *p*-value < 0.05 was considered statistically significant.

## 3. Results

**Figure 1:**
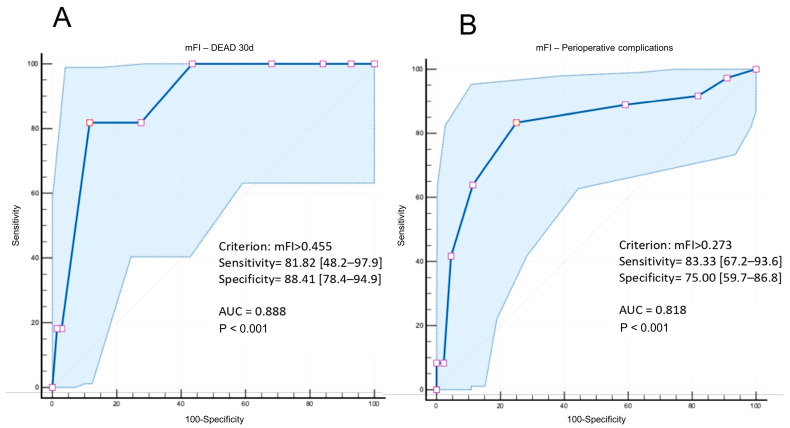
The ROC curve for the modified Frailty Index (mFI) in predicting the 30-day mortality and perioperative complications. (**A**): The optimal cut-off value was identified at mFI > 0.455 for 30-day mortality, providing the best balance between sensitivity and specificity. (**B**): The optimal cut-off value was identified at mFI > 0.273 for perioperative complications, providing the best trade-off between sensitivity and specificity.

Frailty assessment using the mFI demonstrated a good prognostic performance for both short-term outcomes and perioperative complications. For 30-day mortality, the ROC curve analysis identified an optimal mFI threshold of 0.455, yielding a sensitivity of 81.8% (95% CI: 48.2–97.7), specificity of 88.4% (95% CI: 78.4–94.9), and an area under the curve (AUC) of 0.888 (*p* < 0.001) (Figure 1A). For the perioperative complications, the optimal mFI cut-off was 0.273, with a sensitivity of 83.3% (95% CI: 67.2–93.6), specificity of 75.0% (95% CI: 59.7–86.8), and an AUC of 0.818 (*p* < 0.001) (Figure 1B).

Baseline characteristics are shown in Table 1. Table 2 presents intraoperative data and postoperative outcomes.

**Figure 2:**
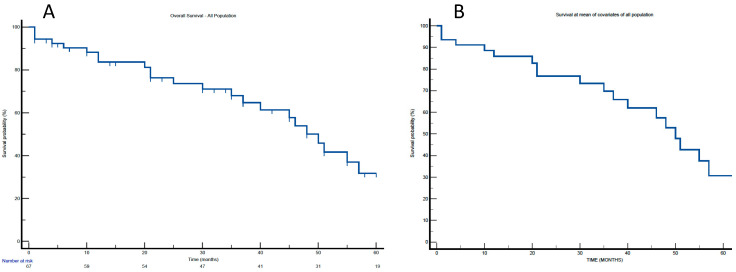
(**A**): Survival curves by Kaplan-Meier. (**B**): Survival curves at a mean of covariates by Cox multivariable proportional hazard regression model.

During a median follow-up of 51.8 ± 36.4 months (range 0–82 months), 24 of 67 patients (35.8%) died. Kaplan-Meier survival analysis (Figure 2A) demonstrated survival rates of 91.0% at 1 month, 83.7% at 12 months, and 31.8% at 57 months.

Multivariable Cox regression analysis for 30-day mortality (Table 3) identified mFI ≥ 0.455 as the strongest independent predictor (HR 6.34; 95% CI: 2.83–8.31; *p* = 0.001).

Additional predictors included heart failure with a preserved ejection fraction (HFpEF) and NT-proBNP > 3.000 pg/mL (HR 5.02; *p* = 0.002), postoperative low-cardiac-output syndrome (LCOS) (HR 4.28; *p* = 0.001), perioperative stroke (HR 3.47; *p* = 0.021), and a minimally invasive thoracotomy (MT) approach (HR 1.84; *p* = 0.049). Adjusted survival probabilities over time, accounting for covariates included in the model, are depicted in Figure 2B.

For long-term mortality (Table 4), significant independent predictors included mFI ≥ 0.273 (HR 6.45; *p* < 0.001), preoperative chronic kidney disease (HR 3.25; *p* = 0.023), and HFpEF with NT-proBNP > 1000 pg/mL (HR 5.33; *p* < 0.001). These findings underline the persistent prognostic value of frailty and comorbid conditions on late outcomes.

## 4. Discussion

This study highlights the prognostic significance of frailty, as measured by the mFI, in a homogeneous cohort of octogenarians undergoing minimally invasive AVR. Our findings demonstrate that the mFI is a reliable predictor of both early mortality and perioperative complications, providing valuable support for its integration into preoperative risk stratification in elderly surgical candidates. In particular, an mFI threshold ≥ 0.455 was the strongest independent predictor of 30-day mortality, while a lower cut-off ≥ 0.273 was associated with perioperative complications. These findings are of particular relevance in a population frequently considered high-risk or marginal for surgery based solely on chronological age, as the identified cutoffs provide an objective framework for recognizing vulnerable patients—an aspect of critical importance in settings with limited resources or in borderline cases between SAVR and TAVI.

The mFI offers several advantages in the surgical setting. It is based entirely on routinely collected clinical variables, without the need for performance-based testing or laboratory measures, and can be readily applied in both retrospective and prospective contexts. Its simplicity makes it particularly suitable for urgent scenarios, high-volume centers, or institutions without structured geriatric co-management. Moreover, the standardized nature of the mFI minimizes inter-observer variability, promoting consistent frailty assessment and facilitating structured, multidisciplinary decision-making within the Heart Team. Despite its simplicity, the mFI has been consistently validated across various surgical disciplines [16,17], and our results reinforce its applicability to cardiac surgery, specifically in minimally invasive AVR.

The concept of futility—defined as the failure to achieve meaningful clinical benefit despite procedural success—has gained prominence in the context of valvular interventions in the elderly. Although the TAVI has expanded therapeutic options for older adults, accumulating data suggest that a subset of patients derive limited benefit. For example, in the PARTNER trial cohort, nearly 20% of patients experienced poor outcomes at 6 months—defined as death or a Kansas City Cardiomyopathy Questionnaire (KCCQ) score < 45—despite technically successful procedures [7]. Similarly, in a large real-world registry of over 2000 patients, approximately 30% had minimal or no symptomatic improvement following the TAVI, with the poorest outcomes observed in those with baseline frailty, advanced disability, or low baseline functional status [6]. These findings underscore the importance of refined risk models that go beyond conventional scoring systems and incorporate frailty as a core variable.

Frailty has thus been recognized as a powerful, independent predictor of poor outcomes following both TAVI and SAVR. The FRAILTY-AVR study by Afilalo et al. demonstrated that the Essential Frailty Toolset (EFT) strongly correlates with 12-month mortality and disability across both procedural strategies [12]. While the EFT has demonstrated high discriminative power, it includes performance-based and biochemical components that may limit its practical use in certain settings. In contrast, the mFI is derived solely from clinical data that are typically available preoperatively, making it more feasible for consistent application in surgical workflows.

Additional insights come from the recent systematic review by Prendiville et al., which confirmed the predictive utility of the Clinical Frailty Scale (CFS) across TAVI and SAVR populations [20]. Their analysis showed that frailty significantly increases the risk of 12-month mortality, yet surgical AVR remained a viable option in selected frail patients, further supporting the notion that frailty should guide—not exclude—treatment decisions.

Compared with tools such as the CFS, which rely on subjective clinical judgment, the mFI offers a more standardized and reproducible approach. This objectivity is particularly advantageous in multicenter studies or when the assessment of frailty is delegated to non-geriatric specialists. The ability to stratify patients using mFI thresholds could also assist in personalizing perioperative care pathways, resource allocation, and shared decision-making with patients and families [14,16].

Our findings are also in line with recent long-term data supporting frailty-based treatment strategies in elderly patients with severe AS. Esposito et al. demonstrated that tailoring interventions according to the frailty severity—using the FIMS score—significantly improved survival and preserved the functional status over a six-year follow-up [21]. In particular, patients with a pre-frail status undergoing TAVR or SAVR had markedly better outcomes than frail patients treated conservatively. These results reinforce the importance of structured frailty assessment—such as the mFI—as a key tool for identifying patients who are most likely to benefit from surgical treatment and for avoiding potentially futile interventions in those with advanced frailty.

Despite the increasing recognition of frailty as a determinant of surgical outcomes, its routine use in cardiac surgery remains limited. Current ESC and AHA/ACC guidelines advocate for the incorporation of frailty assessment into Heart Team discussions but do not specify the preferred tool or implementation strategy [22,23]. Our findings contribute to this evolving field by demonstrating that the mFI is not only feasible but also clinically meaningful in elderly patients undergoing AVR.

Survival data from our study further support the appropriateness of surgery in this population: a 1-year survival rate of 94.3% and a median survival exceeding four years attest to the durability and benefit of surgical AVR when patients are appropriately selected. These results challenge the notion that an advanced age should be a contraindication to surgery, particularly when frailty is assessed and taken into account.

In conclusion, our study reinforces the prognostic value of the modified Frailty Index in elderly patients undergoing minimally invasive AVR. The mFI is a practical, reproducible, and easily applicable tool that can enhance surgical risk assessment and help identify patients most likely to benefit from intervention. Future research should focus on comparative validation of frailty instruments such as the mFI, CFS, and EFT to better define their respective roles across different clinical settings and patient populations.

Despite the encouraging results of our study, several limitations should be taken into account when interpreting the findings. Although the analysis was retrospective in nature, it was based on data collected within a multicenter registry, which contributes to the robustness and external validity of the dataset. However, the number of patients included in this sub-analysis remains limited, and the population was highly selected, consisting exclusively of octogenarians undergoing minimally invasive AVR. This may reduce the generalizability of the results to broader or more heterogeneous populations. Moreover, although the multicenter design adds value in terms of a diversity of practice settings, potential inter-institutional variability in perioperative management and follow-up protocols cannot be entirely excluded. Future studies involving larger, unselected cohorts and including comparisons across centers are warranted to validate our findings and refine frailty-based risk stratification models in the elderly undergoing aortic valve interventions.

## 5. Limitations

This study has several limitations that should be acknowledged. First, its observational and retrospective design introduces the potential for selection bias and confounding. As a retrospective analysis, it is inherently limited in its ability to establish causal relationships, and unmeasured confounders may have influenced the observed associations. Second, the sample size was relatively small, which may limit the statistical power to detect significant differences in survival or complication rates. This limitation is particularly relevant in subgroup analyses, where effect estimates should be interpreted with caution. Third, while the mFI was used as a validated tool for assessing frailty, it may not fully capture the multidimensional nature of frailty, and its optimal cutoff was derived from the same dataset, potentially introducing internal bias. Although the data were drawn from a multicenter registry, this analysis focused on a selected subgroup of very elderly surgical patients, which may limit the generalizability of the findings to broader populations, including those treated with TAVI or deemed inoperable. Finally, long-term follow-up data were obtained through a combination of in-hospital visits and telephone/email contact, which may be subject to reporting inaccuracies or loss to follow-up over extended periods. Future prospective, multicenter studies with larger and more diverse populations are warranted to validate our findings and to refine the role of frailty indices in guiding treatment decisions in elderly patients with aortic valve disease.

## 6. Conclusions

In conclusion, the mFI represents a practical, reproducible, and easily applicable tool for assessing frailty in elderly patients being considered for minimally invasive AVR. In our study, the mFI demonstrated excellent prognostic accuracy for 30-day mortality, with an AUC of 0.888, outperforming conventional risk stratification methods. These findings reinforce the concept that physiological reserve, rather than chronological age alone, should guide clinical decision-making in this fragile population.

In the context of very elderly patients undergoing minimally invasive AVR, the mFI enabled an objective and standardized quantification of frailty, which was effectively integrated into multivariable models. This not only improved the interpretability of our outcomes but also highlighted the prognostic impact of frailty on perioperative risk and short-term survival.

Given the rising number of elderly patients evaluated for AVR in the transcatheter era, incorporating a validated frailty index such as the mFI into routine preoperative assessment could prove essential. It has the potential to optimize patient selection and procedural planning, particularly when weighing the risks and benefits of surgical AVR versus TAVI in borderline or high-risk cases.

Future prospective, multicenter studies involving larger and more diverse cohorts are warranted to validate these findings, assess the impact of frailty-informed decision-making on long-term outcomes, and support the integration of the mFI into contemporary risk scores. Ultimately, the mFI could serve as an indispensable component of a comprehensive, patient-centered approach to care in structural heart disease.

## Figures and Tables

**Table 1:** Baseline preoperative data.

	*n* = 67
Age [years] (SD)	83.1 (2.8)
Female gender (*n*)	45
BMI [kg/m^2^] (SD)	24.8 (4.4)
BSA [m^2^] (SD)	1.66 (0.32)
Congenital bicuspid valve (*n*)	3
Degenerative valve disease	57
Rheumatic valve disease	6
Endocarditis	1
Aortic Stenosis (AS)	24
Aortic Regurgitation (AR)	10
Aortic Combined (AS + AR)	33
Hypertension	53
COPD	16
Diabetes mellitus	16
Insulin-Dependent Diabetes	8
Dialysis	4
CKD	48
Active Smoker	12
Poor mobility	18
TIA	18
Stroke	7
CVD	15
PAD	16
LVEF (SD)	59.4 (5.2)
LVEF < 35% (*n*)	4
Atrial Fibrillation	25
NYHA Functional Class (SD)	2.43 (0.7)
Pulmonary Hypertension (*n*)	6
REDO	8
EuroSCORE II (SD)	5.9 (3.4)
STS Score (*n*)	4.33 (3.1)
Elective Procedure	61
Critical preoperative status	4
Modified Frailty Index (mFI) > 0.273	33
mFI ≥ 0.455	20
HFpEF (with NT-proBNP > 3.000 pg/mL)	8
HFpEF (with NT-proBNP > 1.000 pg/mL)	25

**Table 2:** Univariate analysis of perioperative data reported for all populations. Relative risk is calculated for postoperative (30 days) mortality in all populations.

	All Patients *n* = 67	HR [95% CI]	*p*-Value
HFpEF (with NT-proBNP > 3.000 pg/mL) (*n*)	8	4.92 [3.56–8.74]	0.011
HFpEF (with NT-proBNP > 1.000 pg/mL)	25	1.98 [1.04–4.25]	0.041
Modified Frailty Index (mFI) ≥ 0.273	33	2.81 [1.75–5.22]	0.035
Modified Frailty Index (mFI) ≥ 0.455	20	5.25 [3.25–9.47]	0.011
Operative time [min] (SD)	212.2 (61.4)		
Cardiopulmonary bypass time [min] (SD)	61.1 (41.4)		
Aortic cross-clamp time [min] (SD)	53.7 (32.6)		
Stented tissue valves implanted (*n*)	43	0.84 [0.50–1.094]	0.184
Stentless tissue valves implanted	15	1.25 [0.78–2.43]	0.715
Sutureless valve implanted	9	1.64 [0.75–2.15]	0.554
Valve size mm (SD)	23.2 (2.1)		
Conversion to full sternotomy (*n*)	2	2.35 [1.04–4.1]	0.043
Paravalvular regurgitation (moderate or severe)	1	2.42 [1.32–4.31]	0.064
Perioperative stroke	2	2.28 [1.54–4.53]	0.045
Postoperative AF	15	1.47 [1.09–2.58]	0.052
Pacemaker implantation	3	1.05 [0.26–3.53]	0.630
Perioperative myocardial infarction	1	2.43 [1.31–5.32]	0.160
Postoperative LOS	1	3.85 [2.35–5.54]	0.036
Postoperative IABP	2	2.93 [1.57–3.46]	0.042
Acute kidney failure	4	1.27 [1.04–3.12]	0.052
Chest tubes drainage (mL/m^2^) (SD)	227.57 (83.6)		
Blood transfusion rates (*n*)	21	1.87 [1.04–2.95]	0.025
Re-exploration for bleeding	2	1.95 [1.24–3.26]	0.037
Wound infections	1	2.54 [1.71–4.33]	0.037
Groin complications			
Infections	2	2.2 [0.08–3.74]	0.057
Lymphoceles	1	1.89 [0.21–3.33]	0.084
Femoral art. Pseudo-aneurysm	1	1.04 [0.51–2.04]	0.092
Pneumothorax/subcutaneous emphysema requiring chest tube	2	1.63 [0.74–2.56]	0.124
Significative pulmonary atelectasis	10	2.3 [1.04–4.05]	0.039
Pleural effusions requiring thoracentesis	5	1.15 [0.44–2.05]	0.124
Ventilation time [h] (SD)	8.08 (7.4)		
Prolonged ventilation support > 12 h (*n*)	5	3.4 [1.82–5.65]	0.042
ICU stay [h] (SD)	18.3 (15.2)		
Prolonged ICU stay > 3 days (*n*)	4	4.2 [2.45–6.53]	0.039
Hospital stay [day] (SD)	8.3 (6.2)		
Prolonged hospitalization stay > 10 days	6	2.3 [1.25–4.31]	0.049
Incisional pain score (1–10 scale)			
at ICU discharge (SD)	4.39 (4.13)		
at hospital discharge (SD)	1.85 (2.43)		
At one-month follow-up	1.37 (1.65)		
30-day mortality (*n*)	6		
30-day cardiac death	4		

**Table 3:** The 30-day mortality analysis by the corrected-Cox multivariable proportional hazards regression model: independent predictors of mortality in the population.

	Whole Population	
	HR (Exp(B)) [95% CI]	*p*-Value
Modified Frailty Index (mFI) ≥ 0.455	6.34 [2.83–8.312]	0.001
HFpEF (withNT-proBNP > 3.000 pg/mL)	5.02 [3.42–6.13]	0.002
Postoperative LCOS	4.28 [2.47–6.72]	0.001
Perioperative Stroke	3.47 [2.64–4.21]	0.021
Minithoracotomy	1.84 [1.06–2.75]	0.049

**Table 4:** Long-term mortality analysis by Cox multivariable proportional hazards regression model: independent predictors of mortality in the population.

	Whole Population (Hospital Discharge Survival *n* = 61)	
	HR (Exp(B)) [95% CI]	*p*-Value
Modified Frailty Index (mFI) ≥ 0.273	6.45 [2.41–9.54]	<0.001
Preoperative CKF	3.25 [2.45–5.27]	0.023
Preoperative HFpEF (with NT-proBNP > 1.000 pg/mL)	5.33 [3.45–8.42]	<0.001

## Data Availability

Data are available on request of the corresponding author.

## Abbreviations

The following abbreviations are used in this manuscript:

AKF	Acute Kidney Failure
AS	Aortic Stenosis
AUC	Area Under the Curve
AVR	Aortic Valve Replacement
BMI	Body Mass Index
BSA	Body Surface Area
CKD	Chronic Kidney Disease
CI	Confidence Interval
CFS	Clinical Frailty Scale
COPD	Chronic Obstructive Pulmonary Disease
CT	Computed Tomography
CVD	Cardiovascular disease
EFT	Essential Frailty Toolset
ESC	European Society of Cardiology
E-Value	Expected Value (Sensitivity Analysis)
FIMS	Frailty in Interventional Management Strategy
HFpEF	Heart Failure with Preserved Ejection Fraction
ICU	Intensive Care Unit
IQR	Interquartile Range
KCCQ	Kansas City Cardiomyopathy Questionnaire
LCOS	Low Cardiac Output Syndrome
LOS	Length of Stay
mFI	Modified Frailty Index
MRI	Magnetic Resonance Imaging
MS	Ministernotomy
MT	Mini-thoracotomy
NT-proBNP	N-terminal pro-B-type Natriuretic Peptide
PAD	Peripheral Artery Disease
ROC	Receiver Operating Characteristic
SAVR	Surgical Aortic Valve Replacement
SD	Standard Deviation
SPSS	Statistical Package for the Social Sciences
STS	Society of Thoracic Surgeons
TAVI	Transcatheter Aortic Valve Implantation
TEE	Transoesophageal Echocardiography
TIA	Transient Ischemic Attack
